# Capped silver nanoparticle penetration in primary dentition: an in vitro SEM-EDS analysis

**DOI:** 10.1186/s12903-026-07768-y

**Published:** 2026-02-12

**Authors:** Amjad Almuqrin, Chaminda Jayampath Seneviratne, Jayanti Mendhi, Laurence J. Walsh, Sobia Zafar

**Affiliations:** 1 School of Dentistry, The University of Queensland, 288 Herston Rd, Brisbane, QLD 4006 Australia; 2 School of Dentistry, The University of Hail, Hail, 2440 Saudi Arabia; 3 Central Analytical Research Facility, Queensland University of Technology, Brisbane, QLD 4000 Australia

## Abstract

**Purpose:**

This study investigated the extent and distribution (surface coverage) of penetration of custom-synthesised silver nanoparticles functionalised with two different organic capping agents (casein capped and citrate capped silver nanoparticles).

**Materials and methods:**

Extracted human primary teeth were sectioned into dentine slices, etched to remove the smear layer, and treated topically with either the nanoparticles, silver fluoride (positive control) or deionised water (negative control). Following fixation, dehydration, and carbon coating, samples were examined using a scanning electron microscope (SEM) with backscattered electron detector. The presence of nanoparticles was verified with energy dispersive X-ray spectroscopy (EDS) for spot elemental analysis. Penetration depths were measured from longitudinal-view micrographs, and data were statistically analysed.

**Results:**

All silver nanoparticles treated specimens exhibited evident surface precipitation and intratubular infiltration. Citrate-capped nanoparticles demonstrated greater penetration (32.5 ± 8.9 μm) than casein-capped (19.3 ± 7.1 μm); however, this difference was not statistically significant (*p* = 0.056). In contrast, silver fluoride exhibited significantly deeper and denser infiltration (117.7 ± 29.1 μm), forming continuous intratubular precipitates. EDS confirmed characteristic Ag peaks in all treated samples.

**Conclusion:**

Silver nanoparticles successfully infiltrated dentinal tubules. However, their penetration was shallower than silver fluoride, likely due to lower silver concentration. Their strong surface retention and controlled diffusion indicate potential for use as topical agents. Further optimisation of particle concentration and application protocol may enhance intratubular diffusion while maintaining biological safety.

## Introduction

Cariogenic biofilms develop not only on the superficial surfaces of carious lesions, but also extend through the hard tooth structure, particularly into the dentinal tubules [1]. Once the carious process breaches the dentinoenamel junction (DEJ), microorganisms infiltrate the dentinal tubules, using them as protective microenvironments and pathways toward the pulp. The average length of dentinal tubules is approximately 1.45 mm in primary teeth, and ranges from 1.8 to 2.0 mm in permanent teeth [2]. Tubular diameters vary from 0.9 to 2.5 μm in permanent teeth and from 1.16 to 3.88 μm in primary teeth [3]. Key cariogenic species including *Streptococcus mutans* are capable of invading dentine to mean depths of 500 μm, with maximum penetration exceeding 1,000 μm under optimum in vitro conditions [4]. Moreover, *Candida albicans* colonises dentine *via* hyphal extension, which has been reported to reach up to 184 μm in SEM micrographs [5]. The depth of microbial penetration underscores the need for antimicrobial agents capable of infiltrating sufficiently into dentinal tubules to achieve an effective and sustained caries arrest.

Silver fluoride (SF) is highly effective for caries arrest owing to high silver content, potent fluoride remineralising action, and the capacity to infiltrate dentinal tissue, particularly when demineralised. Demineralisation enlarges dentinal tubules by removing mineral barriers and exposing collagen, thereby creating open pathways for deeper SF penetration [6]. Furthermore, the loss of hydroxyapatite reduces structural resistance, allowing silver to migrate freely through capillary action and fluid diffusion [7]. Once silver ions are inside, they interact with collagen and hydroxyapatite, to form silver-protein complexes and silver phosphate, both of which contribute to the antimicrobial and remineralising effects [8].

Studies have demonstrated that 38% solutions of SF can precipitate silver compounds to depths of up to 1,750 μm in demineralised dentine, and 1,150 μm in sound samples [9]. In another report, silver penetration reached up to 2,490 μm [10]. The majority of silver ions precipitate within minutes following application, while smaller residual amounts may continue to precipitate gradually over time [11]. The extent of discolouration corresponds closely with silver distribution, reflecting its sustained presence in the tissue. While these properties support the efficacy of SF, they also result in its aesthetic drawbacks [12].

The physiochemical transformations that occur in the carious lesion after SF application are not yet fully understood. A recent case report demonstrated that SF followed by stannous fluoride leads to the development of a dense nanosilver-rich surface crust on carious primary molars within three weeks [13]. SEM images have revealed dentine like structures that were free from bacteria, while EDS analysis confirmed the presence of nanoscale silver deposits. This indicates that silver ions undergo in situ reduction within the dentine tubules, forming silver nanoparticles (NPs) spontaneously on SF treated dentine. The emerging evidence thus warrants further investigation of customised metal NPs as potential topical antimicrobial agents for caries arrest.

In previous work, silver and selenium NPs were chemically synthesised and stabilised with natural derived capping agents [14]. These NP formulations are expected to exhibit potent antimicrobial activity while potentially avoiding the tooth discolouration commonly associated with ionic silver compounds. They could replace or augment ionic silver in SF. However, several critical research stages must be completed before their clinical translation can be justified, including evaluation of their penetration into dentine tubules. This study aimed to assess the extent and distribution pattern of casein and citrate capped silver NPs infiltrating primary dentine slices using SEM. The null hypothesis was that there is no significant difference in penetration depth or distribution between casein-capped and citrate-capped silver NPs, nor between the NPs formulations and SF.

## Methods

### NPs preparation

The NPs synthesis process and the physiochemical characterisation were previously described [14]. The Ag1 (casein-capped) and AgL1 (citrate-capped) silver NPs were characterised using UV–visible spectroscopy, transmission electron microscopy (TEM), and inductively coupled plasma optical emission spectroscopy (ICP-OES). TEM analysis confirmed that both Ag1 and AgL1 NPs were predominantly spherical in morphology. The Ag1 exhibited a mean core diameter of 3.6 ± 3.4 nm, while AgL1 had a mean diameter of 3.92 ± 0.71 nm. Elemental analysis by ICP-OES quantified the silver concentrations as 57.44 µg/mL for Ag1 and 121.6 µg/mL for AgL1, with no detectable contamination from heavy metals, confirming the purity of both formulations.

### Dentine slices preparation

Ethical approval for collection of primary teeth was obtained from the Human Research Ethics Committee at The University of Queensland (number: 2024/HE000853). A total of 24 sound extracted primary teeth were exposed to gamma irradiation at 25 kGy for sterilisation purposes [15]. The preparation of dentine slices involved using a low-speed straight handpiece (T1 LINE, Sirona, Bensheim, Germany) with a 22 mm diameter diamond-coated disc (model 6911 H-180, Hyperflex series; Komet Dental, Lemgo, Germany). The slices were perpendicular to the dentinal tubule orientation to maximise tubule exposure and optimise penetration analysis. The smear layer was removed via etching with 37% phosphoric acid gel (Super Etch™, Henry Schein Inc., Melville, NY, USA) followed by rinsing with deionised water and air drying. Thereafter, the dentine slices were divided into four groups (*n* = 6 in each): Ag1, AgL1, SF positive control (Riva Star Aqua™; SDI Limited, Bayswater, VIC, Australia), and deionised water as the negative control. Sample size was determined based on comparable studies [9, 16]. The silver NPs comprised Ag1 (casein-capped silver NPs) and AgL1 (citrate-capped silver NPs). The NP synthesis process was previously described [14].

**Fig. 1:**
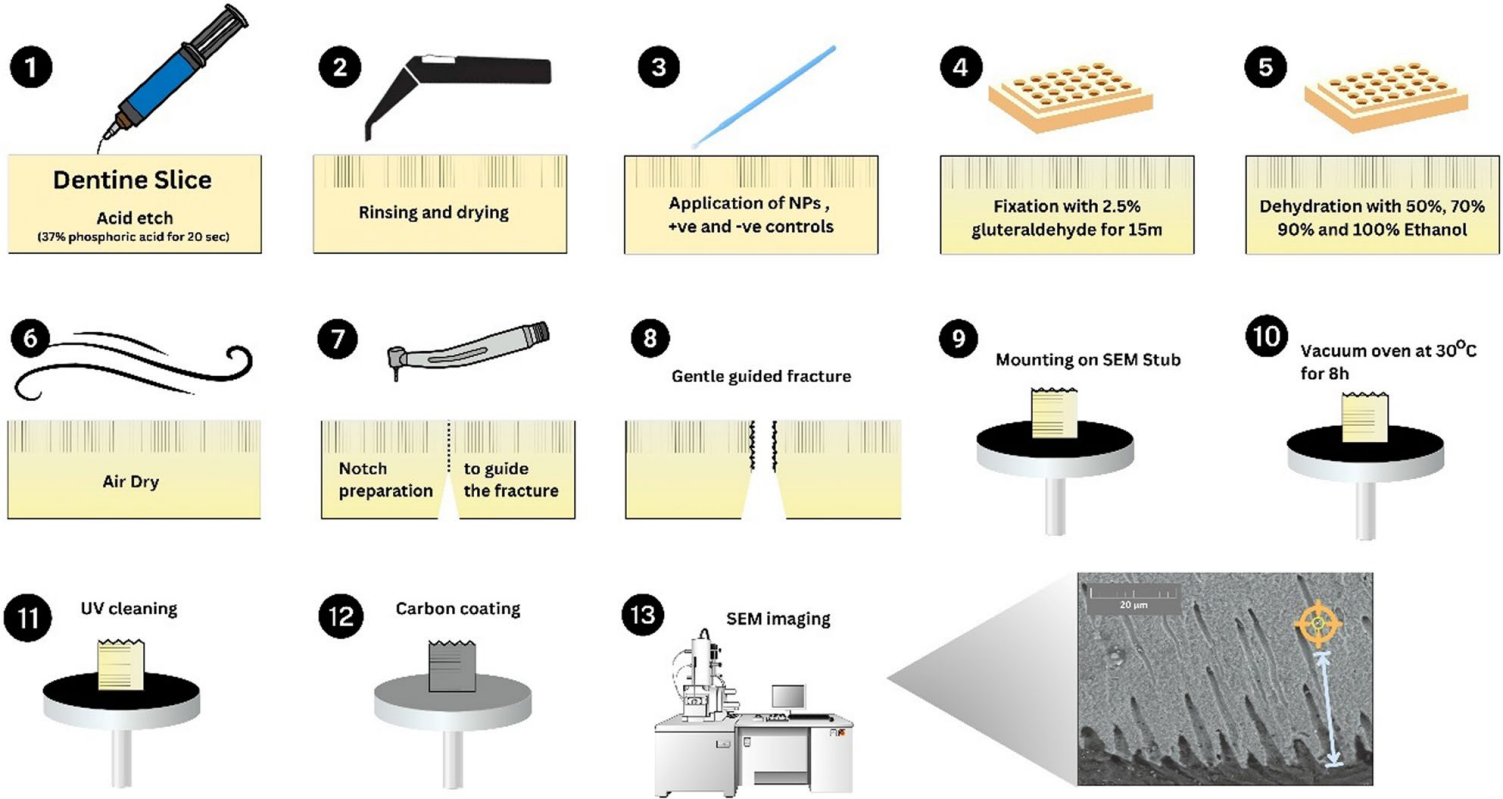
Schematic workflow outlining the sample preparation steps for SEM imaging

**Fig. 2:**
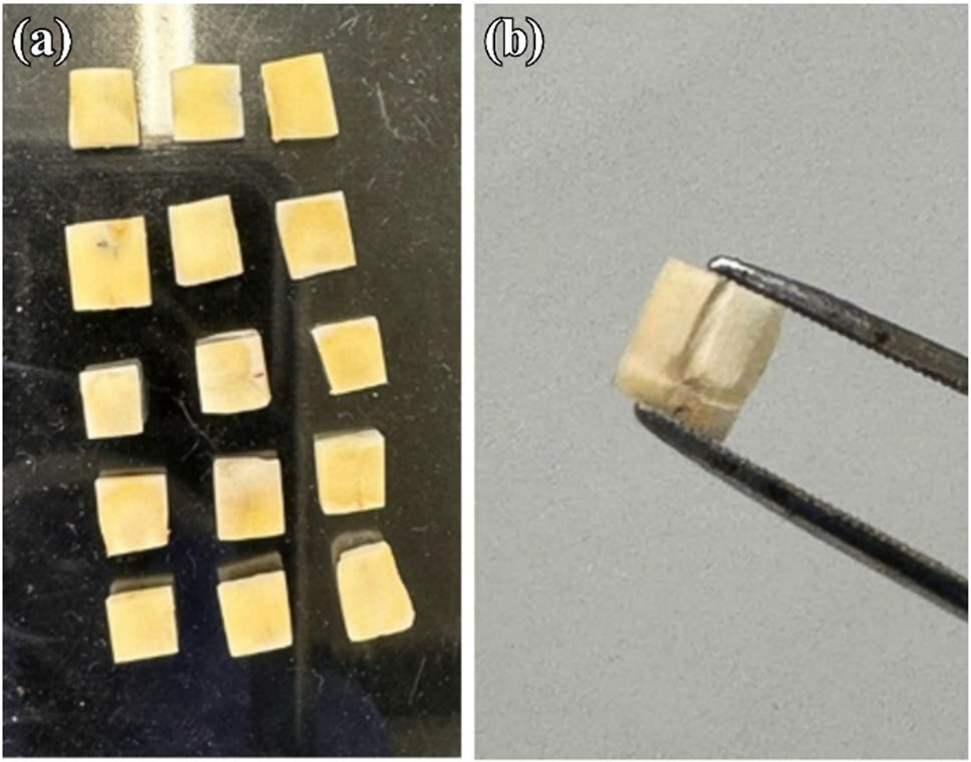
Preparation of dentine slices for SEM analysis. (**a**) Representative image showing dentine slices sectioned from primary teeth, displaying uniform dimensions and flat surfaces. (**b**) A single dentine slice showing the superficial groove prepared at the base to guide the fracture of the sample

One layer of the test solutions was applied to the slices with a microbrush (~ 2 µL) and remained on the dentine surface for approximately 50 s until visibly dry prior to fixation. The specimens were fixed in 2.5% glutaraldehyde (UNILAB, Ajax Finechem, ThermoFisher Scientific, Australia) for 15 min before undergoing sequential dehydration in graded ethanol concentrations (50%, 70%, 85%, and 100%). Afterwards, a superficial longitudinal groove was created at the base of each dentine slice to facilitate controlled fracture to expose dentinal tubules for imaging. The fractured samples were then mounted to aluminium SEM stubs using carbon tape. The samples underwent vacuum oven drying at 30 °C for eight hours before UV cleaning (ZONE II for SEM, Model ZON-2010, SUNYOU, Japan) for five minutes followed by carbon coating (Q150T ES, Quorum Technologies, Lewes, United Kingdom). Secondary emission and backscatter SEM images were acquired using a Tescan MIRA 3 SEM operated at an accelerating voltage of 10–15 kV. Elemental analysis was performed using a ThermoScientific™ UltraDry EDS Detector and processed with Pathfinder software. Sample preparation steps are illustrated in Figs. 1 and 2.

Micrographs were processed using ImageJ software (version 1.54 g; National Institutes of Health, Bethesda, MD, USA) to measure penetration depth. A total of 30 measurements were obtained for each test group, with each measurement taken from a separate dentinal tubule. Penetration depth measurements were performed by drawing a digital line from using ImageJ software by a trained operator. The lines were taken along individual dentinal tubules from the treated surface to the deepest clearly identifiable intratubular silver deposition visible on backscattered electron images. Only intratubular particles were included in the analysis, as particles located around the dentinal tubules were shattered during guided fracture. Data were analysed using Prism version 10.4.1 (GraphPad Software, San Diego, CA, USA). A non-parametric one-way ANOVA (with Dunn’s test) was performed for multiple group comparisons. P-values < 0.05 were considered statistically significant.

## Results

**Fig. 3:**
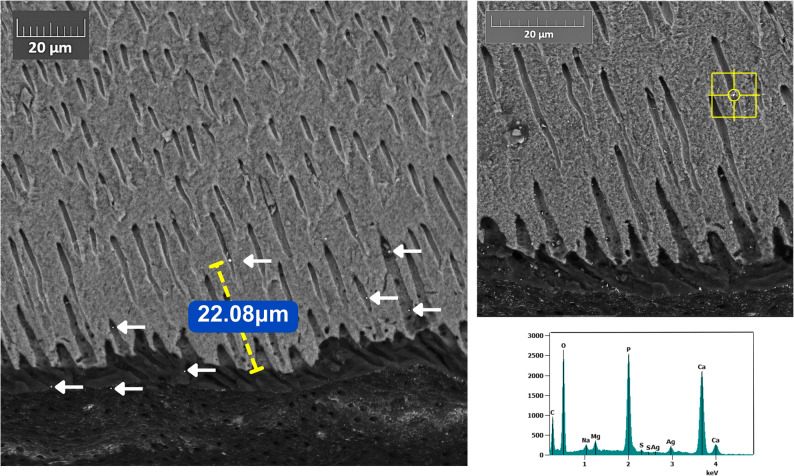
SEM–EDS image of dentine slice treated with Ag1 showing silver penetration of 22.08 µm and localised Ag peaks. Arrows indicate silver nanoparticles

**Fig. 4:**
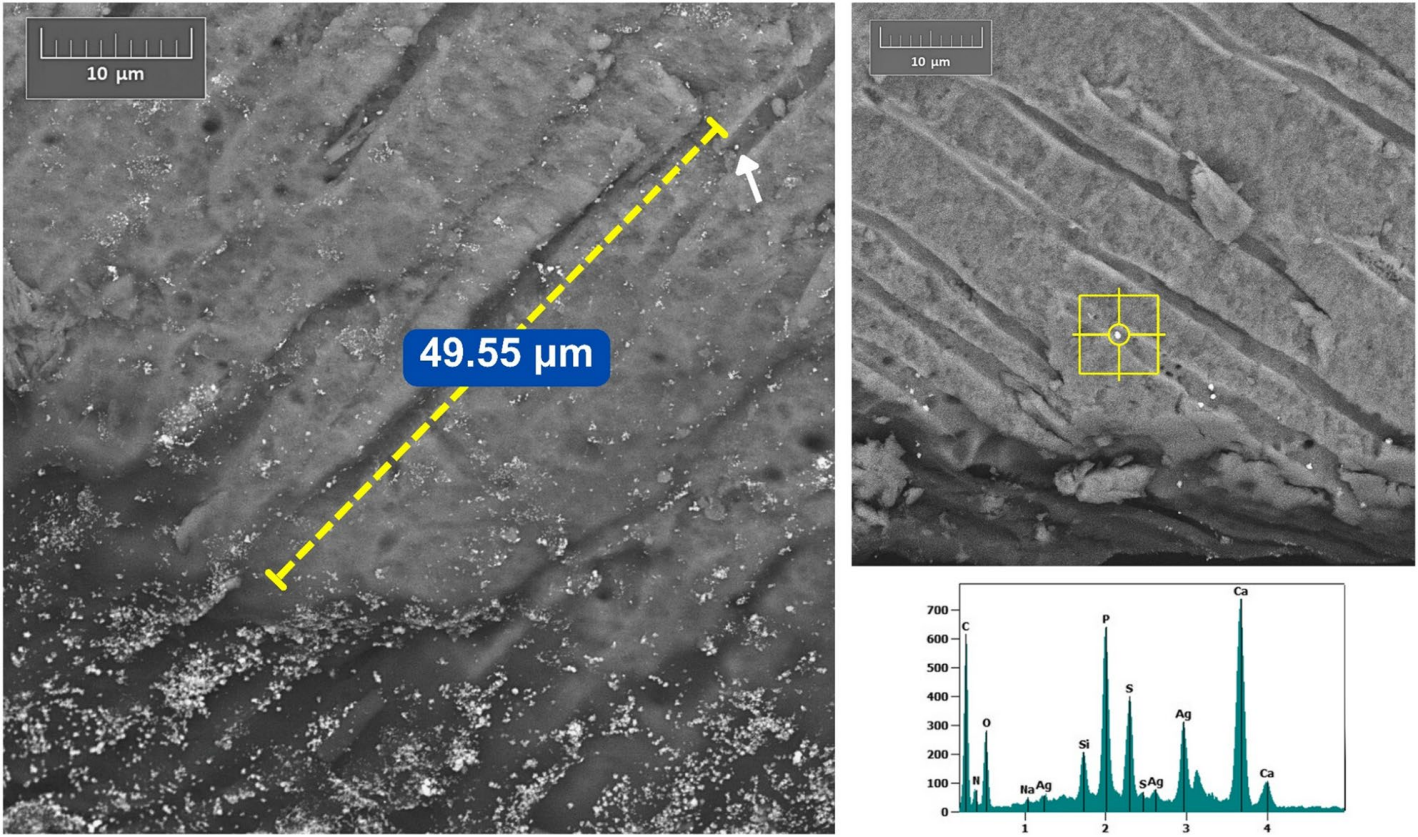
SEM–EDS analysis of dentine treated with AgL1 showing ~49.55 µm penetration and confirmed with distinctive Ag peaks in elemental spot analysis

**Fig. 5:**
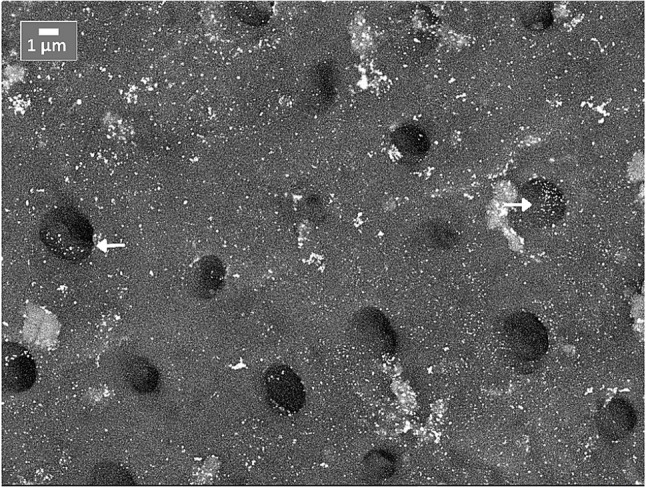
SEM application surface images showing the surface distribution of AgL1 on treated dentine. Arrows indicate nanoparticles located within dentinal tubule orifices

SEM backscatter images showed marked deposition patterns of silver across the experimental groups. Both NP formulations (Ag1 and AgL1) showed electron-dense silver deposits retained on the dentine surface without signs of dissolution or detachment (Figs. 3, 4 and 5) and (Appendix-Figure 8). The citrate-capped AgL1 NPs formed denser, more frequent clusters that extended into the dentinal tubules, whereas the casein-capped Ag1 particles were smaller and more sparsely distributed. In contrast, SF produced continuous, compact electron-dense precipitates that completely sealed the tubules and created a uniform subsurface layer.

**Fig. 6:**
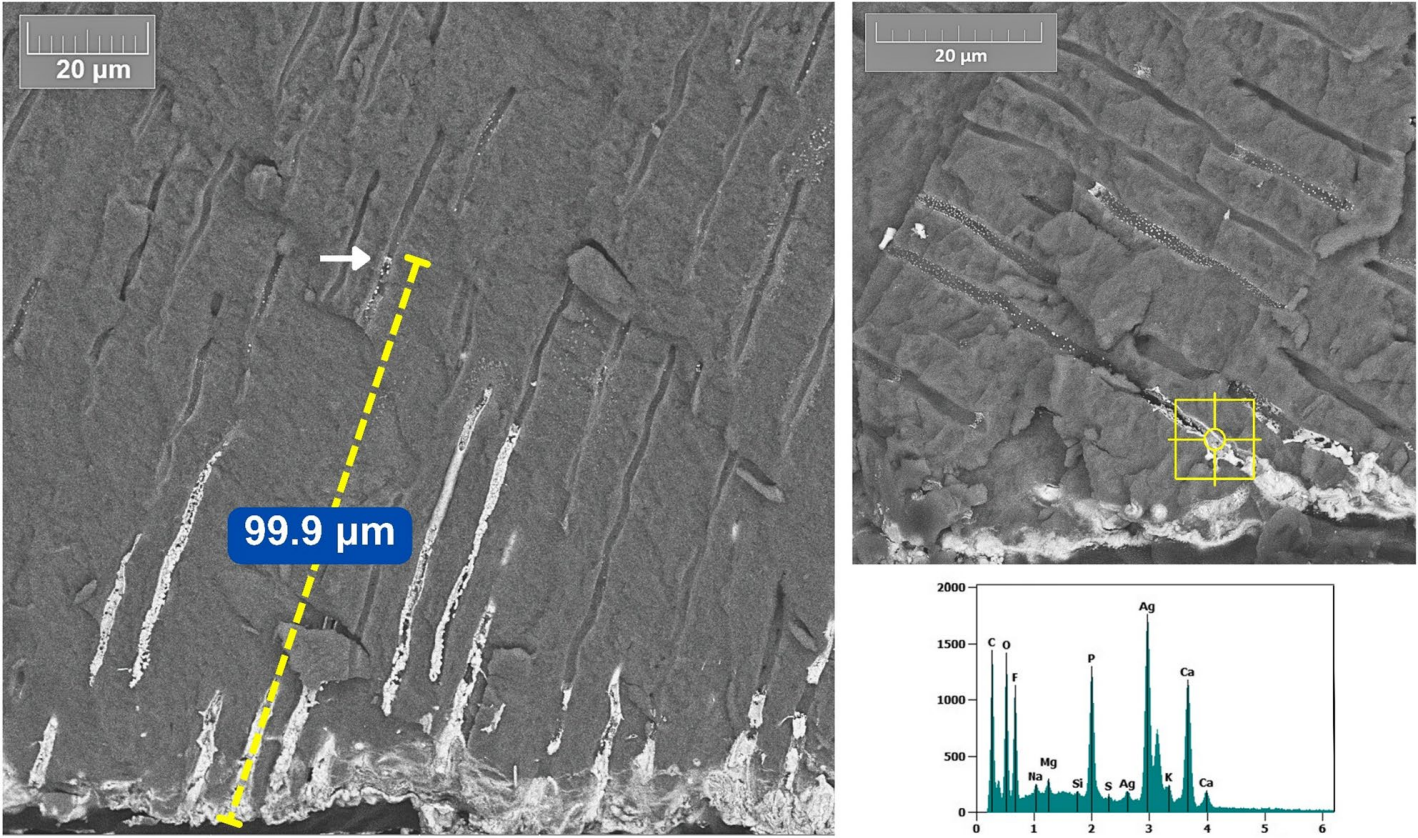
SEM–EDS analysis of dentine treated with SF. Silver penetration along dentinal tubules reached 99.9 µm. EDS confirmed intratubular silver deposition with characteristic Ag peak

**Fig. 7:**
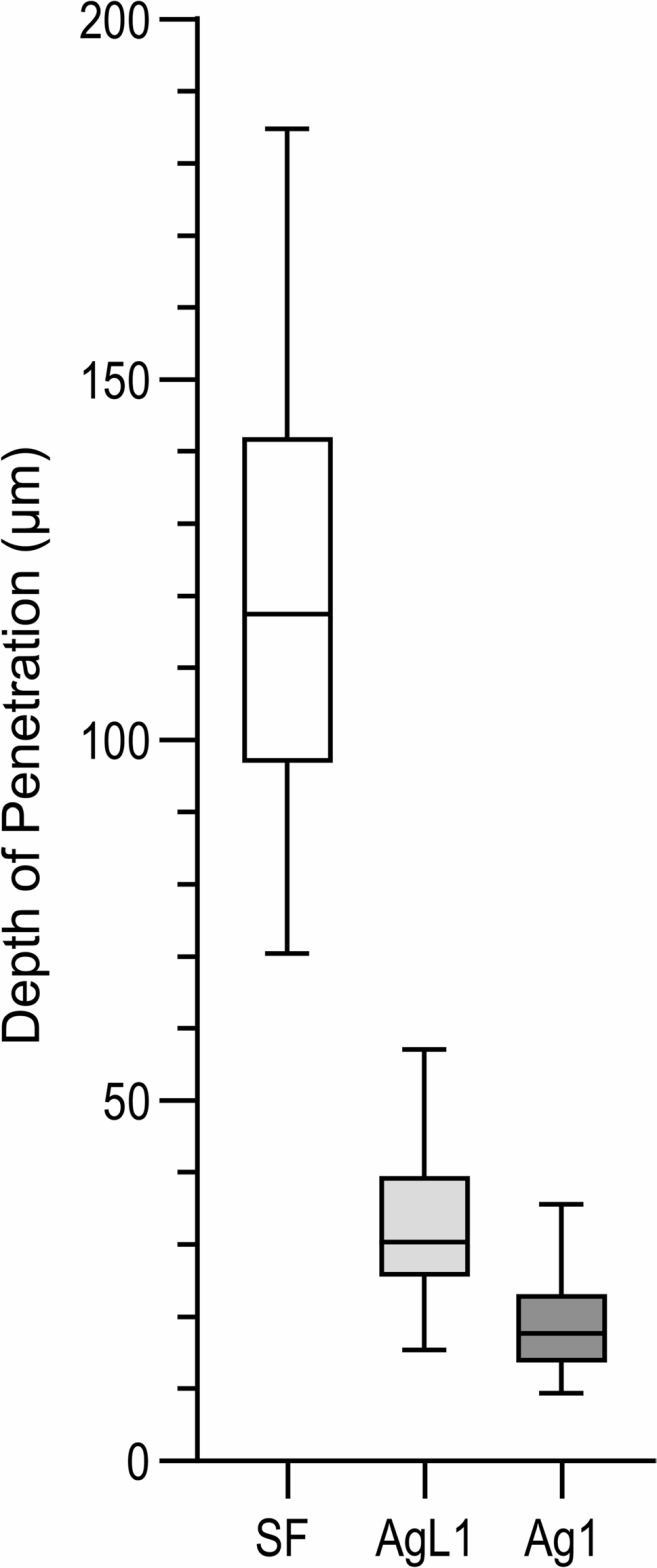
Box-and-whisker plot showing the depth of penetration (µm) of NPs and SF

**Fig. 8:**
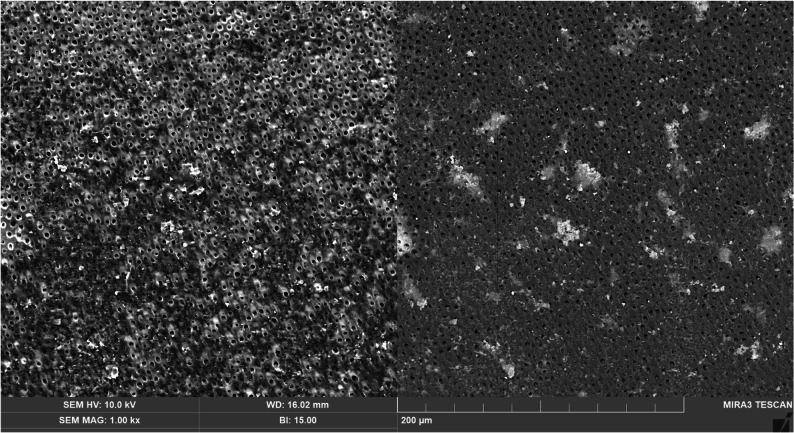
SEM application surface images showing the surface distribution of Ag1 on treated dentine

**Fig. 9:**
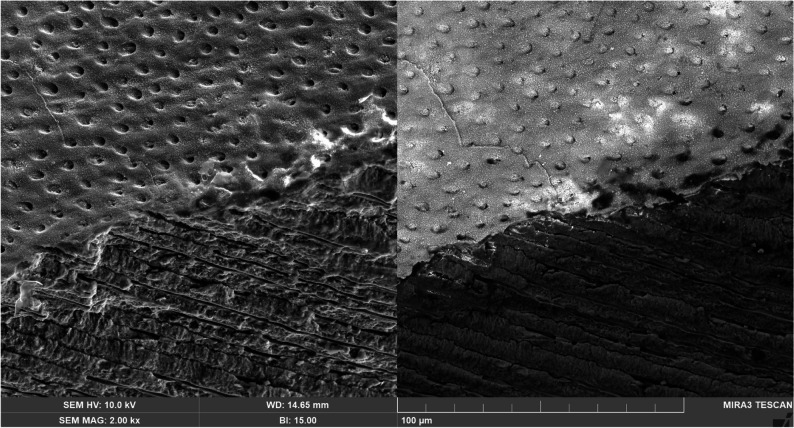
SEM application surface images showing the surface distribution of SF on treated dentine

Quantitatively, the average penetration of AgL1 (32.5 ± 8.9 μm) was slightly greater than Ag1 (19.3 ± 7.1 μm), while SF penetrated to a mean depth of 117.7 ± 29.1 μm (Fig. 6) and (Appendix- Fig. 9**)**, giving a hierarchy of SF > AgL1 > Ag1 in tubular infiltration. The data for penetration is given in Tables 1, 2 and 3; Fig. 7.

EDS verified the presence of silver in the electron-dense areas of all treated specimens through characteristic Ag peaks, thereby confirming that the bright intratubular and surface deposits observed in the SEM micrographs corresponded to silver NPs or ionic silver precipitates.

**Table 1:** Penetration depth (µm) of silver NPs and SF

Group	*n*	Mean ± SD (µm)	Range (µm)	Std. Error of Mean
SF	30	117.7 ± 29.1	70.4–184.8	5.31
AgL1	30	32.45 ± 8.85	15.4–57.1	1.62
Ag1	30	19.32 ± 7.09	9.45–35.6	1.29
DI water	30	(no Ag detected)	—	—

**Table 2:** Results of Dunn’s multiple comparisons test showing pairwise differences in penetration depth among treatment groups

Dunn’s multiple comparisons test	Mean rank diff.	Significant?	Summary	Adjusted *P* Value
SF vs. AgL1	33.70	Yes	***	0.0008
SF vs. Ag1	56.30	Yes	****	< 0.0001
AgL1 vs. Ag1	22.60	No	ns	0.0560
AgL1 vs. DI water	56.30	Yes	****	< 0.0001
Ag1 vs. DI water	33.70	Yes	***	0.0008

*ns *not significant

*** *p* < 0.001; **** *p* < 0.0001

## Discussion

This study is, to the best of the authors’ knowledge, the first to compare two custom-made, organically capped silver NPs formulations with a commercially available 38% SF product, evaluating their penetration in primary dentine. Importantly, these NPs formulations were comprehensively characterised in a prior study [14], with particle size, morphology, and elemental composition, enabling interpretation of dentinal penetration behaviour in the context of known physiochemical properties.

The findings of this in vitro study provide insights into the diffusion behaviour of custom-made silver NPs when applied onto dentine. All NPs treated samples exhibited a uniformly distributed layer that covered the dentine surface. Applicatoin surface micrographs further revealed intratubular infiltration of NPs, which is an important attribute for potential topical anticaries agents. The NPs remained adherent to the dentine throughout sample processing, showing no evidence of detachment, dissolution, or surface degradation, which indicates strong chemical stability on dentine. Longitudinal sections confirmed that both NPs formulations successfully entered the dentine tubular network, with the citrate-stabilised NPs demonstrating slightly greater, but statistically insignificant, intratubular penetration than the casein-capped NPs. This difference may be attributed to variation in particle size and surface charge associated with the capping agents. The null hypothesis was rejected for comparisons between silver NPs and SF, as SF demonstrated significantly greater penetration, but was not rejected for the comparison between casein- and citrate-capped silver NPs.

The reduced visibility and apparent scarcity of NPs observed in Ag1 group **(**Fig. 7**)** can be explained by two principal factors. First, the silver NPs concentration in Ag1 (57.4 µg/mL) was substantially lower than that of the AgL1 (121.6 µg/mL), resulting in fewer detectable particles. Second, the Ag1 NPs were smaller in size (3.6 ± 3.4 nm) compared with those in the AgL1 group (3.92 ± 0.71 nm), which further limits their detectability under SEM due to reduced backscatter contrast. Accordingly, these particles appear less readily visible. Nevertheless, Ag1-derived silver was detectable both on the application surface and within dentinal tubules. By contrast, **(**Fig. 4**)** shows a greater apparent abundance of NPs compared with Ag1. However, detection of these NPs required the use of higher magnification and careful adjustment of sample orientation relative to the fracture plane, which resulted in minor variations in image appearance.

The SF control, which contains ionic silver, produced a denser surface deposit and penetrated substantially deeper than either NPs formulation. This is consistent with the marked difference in silver concentration between SF (266,477 ppm) [17] and both Ag1 and AgL1 (57.44 and 121.6 ppm respectively) [14]. This concentration disparity is reflected in the SEM images, where higher silver content generated stronger backscatter signal and more prominent surface deposits. Additionally, limitation in SEM detection should be considered. Small NPs produce weak backscatter signals and low contrast relative to the surrounding substrate [18]. Consequently, even though NPs may be present within the tubules, their extremely small size and lower atomic interaction volume can render them less detectable using SEM compared with the larger, denser silver precipitates formed by SF.

The ability of NPs to diffuse into dentine tubules is influenced by their physiochemical properties, such as particle size, charge, morphology, capping agent, carrier medium and exposure time [16]. While data on silver NP penetration into dentine are limited, some insights can be drawn from penetration studies of endodontic sealers with antimicrobial NPs. The reported dentine penetration depths include approximately 600 μm for silver NPs, and up to 362 μm for silver NPs capped with chitosan [19]. Larger capping agents such as chitosan or casein would be expected to lower the penetration depth of silver NPs [16]. Moreover, the longer exposure time used in endodontic studies would likely to facilitate greater penetration of NPs compared to the shorter application used in the current study.

The infiltration distance achieved by the silver NPs formulations in this study should be considered in relation to microbial invasion of dentine. Cariogenic microorganisms have been reported to infiltrate dentinal tubules to substantial depths, with *Streptococcus mutans* reaching mean penetration depths of approximately 500 μm and *Candida albicans* hyphal invasion extending up to 184 μm [4, 5]. In comparison, the detectable penetration depths of the citrate-capped and casein-capped silver NPs observed in this study were shallower. Nevertheless, localised intratubular deposition on the lesion surface may be sufficient to suppress microbial viability, limit further progression, and establish a local antimicrobial source within the dentine.

Repeated applications could potentially enhance NPs penetration to greater depths. Further optimisation of particle size, concentration, applications and exposure time is required to explore this point.

The effects of smear layer removal and avoiding the use of demineralised dentine warrant further clarification. Under in vitro conditions, it is practically impossible to trace SF or silver NPs penetration within dentinal tubules without prior removal of the smear layer. The preparation of dentine slices inevitably results in smear layer formation, which can seal dentinal tubules and thereby hinder free NPs diffusion and compromise standardised penetration analysis. Similarly, demineralised dentine exhibits fused and irregular features at the microscopic level, making it difficult to define clear penetration fronts or apply uniform measurement criteria across samples.

From a clinical perspective, Craig and Shi case report has shown nanoscale silver deposits on extracted primary molar following stannous fluoride application [13]. This may indicate that silver NPs are formed spontaneously through chemical reduction of ionic silver in the oral cavity. However, prior to translation, considerations of NPs’ stability, dosage, and toxicity are essential [12, 14]. Capping is a key step for long term stabilisation as unstable NPs tends to agglomerate and change their physiochemical features [14]. The addressed NPs suspensions in this study were capped with natural sourced compounds to achieve maximum shelf life. With respect to cytotoxicity, de Andrade et al. study demonstrated that AgNPs can penetrate dentinal tubules and localise intratubularly without reaching the pulp, which indicates controlled diffusion and biological safety primarily from limited penetration depth and estimated Ag⁺ release relative to reported cytotoxic thresholds [16]. However, no direct cytotoxicity testing, long-term stability assessment, or clinically defined dosing framework was provided. In the present study, the lower silver concentrations used and the shallow and localised intratubular deposition may indicate reduced silver burden thereby less cytotoxicity. Nevertheless, NP-specific risks related to size, surface chemistry, and ion release kinetics remain to explore. Accordingly, future investigations should establish dosing parameters, assess colloidal and intratubular stability, and incorporate cytotoxicity assays using pulp-derived and oral cell models to outline safe and effective application windows, particularly for paediatric use.

Since the present results are derived from in vitro conditions and are limited by SEM-EDS detection, actual penetration of these NPs may be underestimated. Future studies employing high-resolution imaging, such as Xenon Plasma Focused Ion Beam Scanning Electron Microscope (Xe PFIB-SEM), and longer-term ion-release analyses are needed to assess the persistence of these NPs within the dentinal tubule microenvironment. Moreover, the topical application protocol involved a single, short exposure, which may not reflect clinical scenarios involving repeated applications or longer contact times. Another limitation of this study is the inclusion of a single 38% SF formulation as the positive control. A more comprehensive evaluation would involve comparing multiple SF products across different concentrations to better contextualise the penetration behaviour observed.

## Conclusion

Within the limitations of this in vitro investigation, silver NPs demonstrated strong adhesion to the dentine surface and the ability to infiltrate the dentinal tubules of primary teeth, forming stable deposits. Citrate-capped silver NPs showed greater intratubular penetration than casein-capped silver NPs. Repeated applications may further enhance the diffusion of these NPs into dentinal tubules.

## Data Availability

The datasets generated and analysed during the current study are available from the corresponding author on reasonable request.

## Appendix

**Table 3:** Penetration depth measurements of the NPs and control groups

SF	AgL1	Ag1	DI water
71.817	28.909	20.974	0
134.164	24.387	14.422	0
143.764	25.688	24.832	0
127.633	15.398	22.679	0
86.988	25.262	19.641	0
98.537	40.194	17.44	0
98.664	30.759	15.605	0
184.77	39.789	18.669	0
93.759	20.809	12.476	0
147.499	28.281	15.671	0
141.434	30.068	9.445	0
181.411	36.141	13.152	0
147.887	39.444	12.056	0
118.8	43.858	14.859	0
145.129	39.853	10.855	0
86.636	57.076	13.867	0
127.215	20.555	22.089	0
76.984	30.014	17.931	0
119.295	25.158	19.176	0
120.885	19.881	15.893	0
143.966	26.766	30.045	0
123.695	29.752	33.688	0
116.055	29.146	25.276	0
112.903	33.446	12.211	0
109.403	39.693	21.781	0
115.671	42.104	33.201	0
101.415	38.608	26.813	0
85.99	35.452	12.78	0
70.422	38.054	16.587	0
97.895	38.812	35.619	0

## Abbreviations

Ag: Silver
Ag1: Casein-capped silver nanoparticles
AgL1: Citrate-capped silver nanoparticles
AgNPs: Silver nanoparticles
ANOVA: Analysis of variance
BSE: Backscattered electrons
Ca: Calcium
CaF₂: Calcium fluoride
CBSE: Converted backscattered electrons
DEJ: Dentino-enamel junction
DI water: Deionised water
EDS: Energy-dispersive X-ray spectroscopy
EDTA: Ethylenediaminetetraacetic acid
FAp: Fluoroapatite
GSH: Glutathione
HAp: Hydroxyapatite
ICP-OES: Inductively coupled plasma optical emission spectroscopy
kGy: Kilogray
kV: Kilovolt
µg/mL: Micrograms per millilitre
µm: Micrometre
micro-CT: Micro-computed tomography
micro-PIXE: Micro-particle-induced X-ray emission
NPs: Nanoparticles
OM: Optical microscopy
PIGE: Particle-induced gamma-ray emission
ppm: Parts per million
qBSE-SEM: Quantitative backscattered electron scanning electron microscopy
SEM: Scanning electron microscopy
SE: Secondary electrons
SD: Standard deviation
SF: Silver fluoride
SDF: Silver diamine fluoride
TEM: Transmission electron microscopy
UV: Ultraviolet
Xe PFIB-SEM: Xenon plasma focused ion beam scanning electron microscopy
